# The association of depression and sleep disorders in patients with type 1 diabetes in Taiwan

**DOI:** 10.1097/MD.0000000000038969

**Published:** 2024-07-19

**Authors:** Meng-Han Ni, Yi-Sun Yang, Jing-Yang Huang, Shih-Chang Lo, Chien-Ning Huang, Edy Kornelius

**Affiliations:** a School of Medicine, Chung Shan Medical University, Taichung, Taiwan; b Department of Internal Medicine, Division of Endocrinology and Metabolism, Chung Shan Medical University Hospital, Taichung, Taiwan; c Department of Medical Research, Chung Shan Medical University Hospital, Taichung, Taiwan; d Institute of Medicine, Chung Shan Medical University, Taichung, Taiwan.

**Keywords:** depression, diabetes, sleep disorder, type 1 diabetes, type 2 diabetes

## Abstract

The association between depression and sleep disorders in patients with type 1 diabetes mellitus (T1DM) in Taiwan is underexplored. We used a nationwide population-based dataset to evaluate the association of T1DM with these conditions in Taiwan from 2001 to 2019. Patients with T1DM were identified as cases, and 2 control groups were used for comparison: patients with type 2 diabetes mellitus (T2DM) and nondiabetic patients. Age, sex, date of diagnosis, and multiple comorbidities were included and matched using propensity score matching between cases and controls. The primary outcome of this study was to identify new occurrences of the first diagnosis of depression or sleep disorders. After matching, this study included 27,029 T1DM cases, 54,058 T2DM controls, and 108,116 nondiabetic controls. Patients with T1DM exhibited a 1.55-fold higher risk of developing depression (hazard ratio [HR] 1.55, 95% confidence intervals [CI] 1.48–1.61) and a 1.41-fold higher risk of experiencing sleep disorders (HR 1.41, 95% CI 1.37–1.46) compared to nondiabetic controls. Similarly, patients with T2DM displayed elevated risks of both depression (HR 1.39, 95% CI 1.34–1.43) and sleep disorders (HR 1.40, 95% CI 1.37–1.44) relative to non-diabetic controls. When comparing the T1DM and T2DM groups, T1DM patients demonstrated a slightly higher risk of depression (HR 1.11, 95% CI 1.07–1.16) but no significant difference in the risk of sleep disorders compared to T2DM patients. These results were consistent regardless of different ages or sexes. This study demonstrates a significant association between diabetes mellitus and the risk of depression and sleep disorders in a large cohort of Taiwanese patients.

## 1. Introduction

The prevalence of type 1 diabetes mellitus (T1DM) in Taiwan is increasing,^[1]^ particularly affecting young children and persisting as a lifelong condition.^[2]^ Concurrently, the intersection of diabetes and mental health is gaining recognition as a critical area of concern.^[3–7]^ Mental health disorders, particularly depression and sleep disorder, have a documented prevalence in the general population and are known to compromise quality of life and functional capacity.^[8,9]^ In individuals with diabetes, these conditions can significantly impede effective disease management, leading to poorer glycemic control and increased complication rates.^[10–12]^

However, while the link between type 2 diabetes mellitus (T2DM) and mental health issues is well-established,^[13–16]^ the specific challenges and prevalence of such disorders in T1DM have been underexplored.^[17,18]^ This oversight persists despite evidence suggesting that the psychological burden may be even greater in T1DM due to the early onset and lifelong management challenges associated with the disease.^[19,20]^ These psychological burdens can severely affect educational achievement and daily functionality, further complicating the management of their condition.^[11,21–23]^

In response to this critical gap, our study utilizes population-based dataset from the National Health Insurance Research Database (NHIRD) to systematically evaluate the risk of mental disorders among individuals with T1DM in Taiwan. By comparing these individuals with non-diabetic individuals and those with T2DM, this study aims to elucidate the psychological comorbidities associated with T1DM.

## 2. Methods

### 2.1. Data source

This study utilized the Taiwan NHIRD, which was released and audited by the Department of Health and the Bureau of the National Health Insurance Program for scientific research purposes. Taiwan’s National Health Insurance, a mandatory universal health insurance program, was implemented in 1995 and provides comprehensive medical care coverage to nearly 99% of the Taiwanese population. In this study, we analyzed NHIRD dataset from January 1, 2011, to December 31, 2019. The Health and Welfare Data Science Center, part of Taiwan’s Ministry of Health and Welfare, consolidates the NHIRD with other health databases to enhance data management and analytical processes. The NHIRD compiles comprehensive datasheets, including beneficiary registries, ambulatory and inpatient claims, diagnostic codes, pharmacy prescriptions, and medical facility details. The International Classification of Diseases, 9th Revision (ICD-9-CM) and International Classification of Diseases, 9th Revision (ICD-10-CM) coding system for disease diagnoses was employed to identify patients with T1DM and T2DM. All patient credentials and social security numbers were encrypted to ensure privacy and confidentiality. The study protocol was approved by the Institutional Review Board of Chung Shan Medical University Hospital, identified by the Institutional Review Board number CS17153.

### 2.2. Study group

In this study, cases comprised patients diagnosed with T1DM, as determined by clinical doctors during professional medical consultations, utilizing specific diagnostic codes from the ICD-9-CM: 250.x1, 250.x3; ICD-10-CM: E10. For comparison, 2 control groups were selected: patients with T2DM and non-diabetic individuals. T2DM patients were identified through diagnostic codes (ICD-9-CM: 250.xx, excluding 250.x1, 250.x3; ICD-10-CM: E11-E13). The index date was defined as the initial available visit indicating T1DM or T2DM diagnosis within the study period. To ensure the representativeness of the selected cohort, stringent criteria were applied. This included the requirement for both cases and control patients in the database to have attended outpatient departments a minimum of 3 times and to have been hospitalized at least once. For instance, patients with T1DM cases should have had at least 3 outpatient departments visit and at least one admission record. The same criteria were applied to the control groups to maintain consistency and comparability across the study population.

To ensure the accuracy of diabetes duration within this database, patients appearing before 2012 were excluded. This approach enables the identification of newly diagnosed diabetes patients, as those whose first appearance is in 2012 or later are considered as such. By implementing this criterion, we mitigate the inclusion of individuals with preexisting diabetes diagnoses, thus facilitating a more precise assessment of diabetes duration and its associated factors within the study population.

Comorbidities that could potentially influence the study were selected for analysis, including sex, age, urbanization status, insurance type, and yearly income. Additionally, disease comorbidities such as asthma, atopic dermatitis, and chronic conditions like hypertension, diabetes, cardiovascular disease, heart failure, and malignancy were included in the analysis. The rationale behind this comprehensive selection is to account for various factors that may confound the relationship between the primary outcomes of depression and sleep disorders and other variables of interest. By including these comorbidities and baseline medication data from the 6 months preceding the index date, we aim to control for potential confounders and enhance the accuracy and validity of our study findings. Detailed coding of diagnoses and comorbidities for this study is provided in Table S1, Supplemental Digital Content, http://links.lww.com/MD/N236.

### 2.3. Outcome

The primary outcome of this study was to identify new occurrences of first diagnoses of depression (ICD-9: 296, 300, 309, 311; ICD-10: F30-F34, F40-F45) or sleep disorders (ICD-9: 327, 347, 307.4, 770.8, 780.5, V69.4; ICD-10: F51, G47, Z72.8). These diagnoses were obtained during professional medical visits conducted throughout the follow-up period.

### 2.4. Statistical analysis

All data were encrypted and analyzed by an independent statistician. Data analysis was conducted using SAS 9.4 software (SAS Institute Inc., Cary, NC). Descriptive statistics were utilized to summarize the demographic characteristics of the study population, presenting mean and standard deviation for continuous variables, and frequencies and percentages for categorical variables. Propensity score matching (PSM) was employed to match cases and controls. Given the higher incidence rates of depression and sleep disorders in both type 1 DM and type 2 DM patients, we chose a 1:2 matching ratio by age, sex, and diagnosis year to match between type 1 DM patients with type 2 DM patients. Additionally, considering the lower incidence rates of depression and sleep disorders in non-diabetic controls, we chose a 1:4 matching ratio for type 1 DM patients with non-diabetic controls.

For both PSM processes, the ATE of type 1 DM was calculated by logistic regression using the PROC PSMATCH procedure in SAS software. The ATE was calculated using covariates of age, sex, and year of DM diagnosis, and the matching algorithm was conducted using nested greedy matching with a caliper of 0.01. An absolute standardized mean difference (ASD) of <0.1 between the 2 groups after PSM was considered as well-balanced.

Table S2, Supplemental Digital Content, http://links.lww.com/MD/N237 demonstrates that most demographic and socioeconomic characteristics have similar distributions among the 3 groups, with an ASD of <0.1, indicating well-balanced groups. However, it is worth noting that, compared to non-DM individuals, patients with diabetes have slightly higher comorbidities and medication prescription proportions, as indicated by an ASD > 0.1.

The primary outcome variable, the occurrence of first diagnosis of depression or sleep disorders, underwent analysis using survival analysis techniques. Kaplan–Meier survival curves were generated to illustrate the cumulative incidence of depression and sleep disorders over time. The log-rank test was employed to assess differences in survival distributions between subgroups.

To evaluate the association between potential risk factors and the occurrence of depression and sleep disorders, univariate and multivariate Cox proportional hazards regression models were utilized. Hazard ratios (HRs) and 95% confidence intervals (CIs) were calculated to estimate the strength of association between each risk factor and the outcome, while adjusting for potential confounders. All statistical tests were two-tailed, and *P*-values <.05 were deemed statistically significant.

## 3. Results

**Figure 1.**
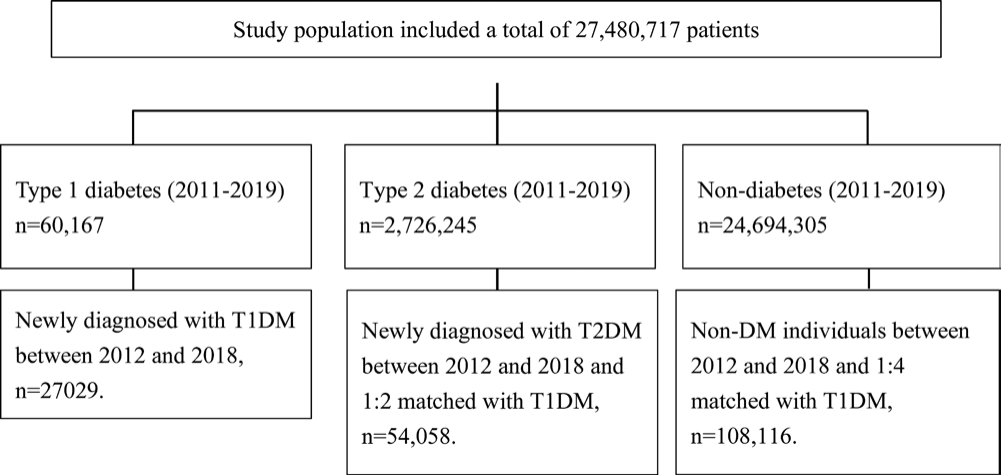
Flow diagram of this study population.

The data analysis encompassed a total of 27,480,717 patients. Prior to matching, the study identified 60,167 new onset cases of T1DM. The T2DM control group comprised 2726,245 patients, while the non-diabetic group consisted of 24,694,305 patients. Following PSM, the cohort was refined to include 27,029 T1DM cases, 54,058 T2DM controls, and 108,116 non-diabetic controls (Fig. 1).

Table 1 presents the baseline characteristics of the matched diabetes population in this study. Across the index years from 2012 to 2018, the distribution of cases remained consistent, demonstrating well-matched cohorts throughout the study period. Sex distribution was balanced, with males accounting for 51.2% and females 48.8% of the cohort. Age distribution also exhibited homogeneity, with a comparable mean age at index across all groups, predominantly falling between 40–64 years old (51%). Urbanization status indicated a majority residing in urban areas (approximately 60%). The prevalence of comorbidities such as hypertension (40%) and hyperlipidemia (28%) was notably higher in diabetic groups, with corresponding medication usage also elevated.

**Table 1:** Baseline characteristics of matched diabetes population in Taiwan.

	T1DM	T2DM	Non-DM
n	27,029	54,058	108,116
Index year			
2012	4602 (17.0%)	9204 (17.0%)	18,408 (17.0%)
2013	4209 (15.6%)	8418 (15.6%)	16,836 (15.6%)
2014	4454 (16.5%)	8908 (16.5%)	17,816 (16.5%)
2015	3874 (14.3%)	7748 (14.3%)	15,496 (14.3%)
2016	4114 (15.2%)	8228 (15.2%)	16,456 (15.2%)
2017	3031 (11.2%)	6062 (11.2%)	12,124 (11.2%)
2018	2745 (10.2%)	5490 (10.2%)	10,980 (10.2%)
Sex			
Male	13,830 (51.2%)	27,660 (51.2%)	55,320 (51.2%)
Female	13,199 (48.8%)	26,398 (48.8%)	52,796 (48.8%)
Age at index	55.7 ± 18.1	55.8 ± 17.8	55.6 ± 18.1
<20	1580 (5.9%)	2933 (5.4%)	6279 (5.8%)
20–39	3483 (12.9%)	7123 (13.2%)	14,058 (13.0%)
40–64	13,740 (50.8%)	27,705 (51.3%)	55,405 (51.3%)
≥65	8226 (30.4%)	16,297 (30.2%)	32,374 (29.9%)
Urbanization			
Urban	15,986 (59.1%)	31,364 (58.0%)	65,850 (60.9%)
Suburban	8296 (30.7%)	16,991 (31.4%)	31,998 (29.6%)
Rural	2747 (10.2%)	5703 (10.6%)	10,268 (9.5%)
Unit type of insured			
Government	837 (3.1%)	1835 (3.4%)	4713 (4.4%)
Privately held company	14,418 (53.3%)	29,387 (54.4%)	58,319 (53.9%)
Agricultural organizations	4674 (17.3%)	9222 (17.1%)	16,752 (15.5%)
Low-income	646 (2.4%)	935 (1.7%)	1479 (1.4%)
Nonlabor force	6053 (22.4%)	11,721 (21.7%)	23,760 (22.0%)
Others	401 (1.5%)	958 (1.8%)	3093 (2.9%)
Insured amount (NTD)			
<20,000	6997 (25.9%)	13,124 (24.3%)	33,125 (30.6%)
20,000–40,000	14,623 (54.1%)	29,200 (54.0%)	50,489 (46.7%)
>40,000	5409 (20.0%)	11,734 (21.7%)	24,502 (22.7%)
Co-morbidities			
Asthma	715 (2.7%)	1317 (2.4%)	1327 (1.2%)
Allergic rhinitis	1258 (4.7%)	2135 (4.0%)	2986 (2.8%)
Atopic dermatitis	1720 (6.4%)	2773 (5.1%)	3373 (3.1%)
Hypertension	10,777 (39.9%)	18,252 (33.8%)	16,224 (15.0%)
Cardiovascular disease	2344 (8.7%)	3817 (7.1%)	3737 (3.5%)
Hyperlipidemia	7579 (28.0%)	8161 (15.1%)	6883 (6.4%)
Heart failure	978 (3.6%)	1215 (2.3%)	1022 (1.0%)
Malignancy	1131 (4.2%)	1874 (3.5%)	2755 (2.6%)
ESRD	1340 (5.0%)	778 (1.4%)	915 (0.9%)
Ischemic stroke	1086 (4.0%)	1490 (2.8%)	1467 (1.4%)
Hemorrhage stroke	208 (0.8%)	279 (0.5%)	344 (0.3%)
ADHD	27 (0.1%)	63 (0.1%)	67 (0.1%)
Autism	5 (0.0%)	47 (0.1%)	9 (0.0%)
Medication			
NSAIDs	11,962 (44.3%)	21,449 (39.7%)	27,182 (25.1%)
Corticosteroids	2941 (10.9%)	5262 (9.7%)	5625 (5.2%)
Anxiolytics			
Benzodiazepines	4436 (16.4%)	6989 (12.9%)	8416 (7.8%)
Azaspirodecanedione	13 (0.1%)	39 (0.1%)	40 (0.0%)
Diphenylmethane	126 (0.5%)	182 (0.3%)	192 (0.2%)
Other anxiolytics	1110 (4.1%)	1869 (3.5%)	1979 (1.8%)
Hypnotics			
Benzodiazepine	1602 (5.9%)	2169 (4.0%)	2541 (2.4%)
Z-drugs	1713 (6.3%)	2508 (4.6%)	3069 (2.8%)
Antidepressants			
SSRI	597 (2.2%)	1069 (2.0%)	1294 (1.2%)
Non-SSIR	581 (2.2%)	704 (1.3%)	639 (0.6%)

ADHD = attention deficit hyperactivity disorder; DM = diabetes mellitus; ESRD = end stage renal disease; NSAID = non-steroidal anti-inflammatory drugs; NTD = New Taiwan dollar; SSRI = selective serotonin reuptake inhibitor; T1DM = type 1 diabetes mellitus; T2DM = type 2 diabetes mellitus; Z-drugs = zaleplon, zolpidem, and eszopiclone.

Table 2 illustrates the risk of new occurrences of depression and sleep disorders within cohorts of T1DM, T2DM, and non-diabetic controls. The incidence rates of both depression and sleep disorders were notably elevated among individuals with diabetes compared to non-diabetic controls. Following adjustment for confounding variables, individuals with T1DM exhibited a 1.55-fold (HR 1.55, 95% CI 1.48–1.61) higher risk of developing depression and a 1.41-fold (HR 1.41, 95% CI 1.37–1.46) higher risk of experiencing sleep disorders compared to non-diabetic controls. Similarly, individuals with T2DM displayed elevated risks of both depression (1.39-fold) with HR 1.39, 95% CI 1.34–1.43 and sleep disorders (1.40-fold) with HR 1.40, 95% CI 1.37–1.44 relative to non-diabetic controls. Interestingly, when comparing T1DM and T2DM groups, T1DM patients demonstrated a slightly higher risk of depression (1.11-fold) with HR 1.11, 95% CI 1.07–1.16 but no significant difference in the risk of sleep disorders compared to T2DM patients.

**Table 2:** Association of depression and sleep disorders among patients with type 1 diabetes mellitus, type 2 diabetes mellitus, and nondiabetic controls.

	T1DM	T2DM	Non-DM
*Depression*			
Person-months	1,049,332	2,241,451	5,075,017
Event	4014	7198	10,095
Incidence rate*	3.83 (3.71–3.95)	3.21 (3.14–3.29)	1.99 (1.95–2.03)
aHR	1.55 (1.48–1.61)	1.39 (1.34–1.43)	Reference
aHR	1.11 (1.07–1.16)	Reference	0.72 (0.70–0.74)
*Sleep disorders*			
Person-months	873,730	1,849,806	4,502,814
Event	5990	11,637	16,978
Incidence rate*	6.86 (6.68–7.03)	6.29 (6.18–6.41)	3.77 (3.71–3.83)
aHR	1.41 (1.37–1.46)	1.40 (1.37–1.44)	Reference
aHR	1.01 (0.97–1.04)	Reference	0.71 (0.70–0.73)

aHR = adjusted hazard ration (adjusted by age, sex, and year of diagnosis), DM = diabetes mellitus, T1DM = type 1 diabetes mellitus, T2DM = type 2 diabetes mellitus.

* Incidence rate, per 1000 person-months.

**Figure 2.**
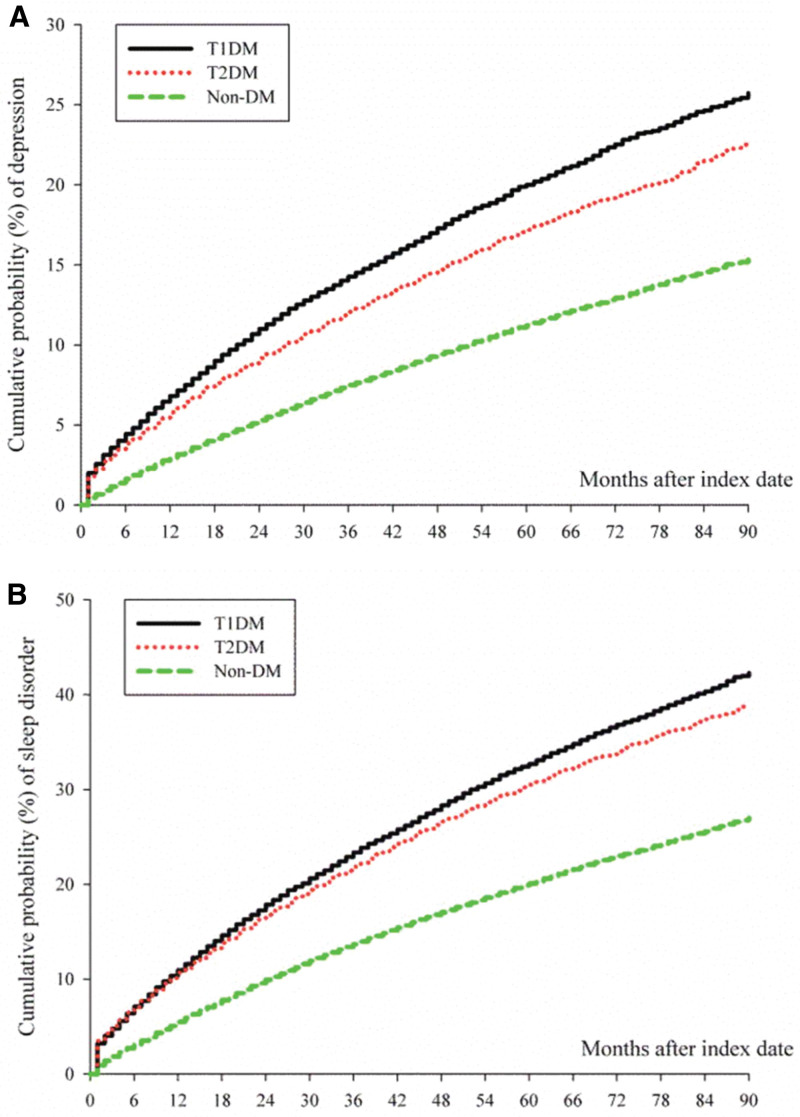
Kaplan–Meier curves evaluating the association between type 1 diabetes mellitus, type 2 diabetes mellitus, and non-diabetic patients. (A) Depression; (B) sleep disorder.

Figure 2A presents a Kaplan–Meier curve comparing the incidence of depression among patients with T1DM, T2DM, and non-diabetic patients. The curve diverges after 6 months of follow-up, indicating a higher risk of depression in both T1DM and T2DM compared to non-diabetic patients. Similarly, Figure 2B displays a Kaplan–Meier curve illustrating the incidence of sleep disorders among the same groups. While the association between T1DM and T2DM is comparable, both groups exhibit a significantly higher risk of sleep disorders compared to non-diabetic patients.

Table 3 presents a subgroup analysis of sex-stratified risk for depression and sleep disorders among individuals with T1DM and T2DM, compared to non-diabetic controls. The findings from this analysis align consistently with the main results, demonstrating that both male and female patients with diabetes exhibit significantly elevated risks of depression and sleep disorders compared to non-diabetic controls. After adjusting for confounding factors, patients with T1DM consistently displayed higher risks of depression compared to those with T2DM and non-diabetic controls, regardless of gender. Specifically, males with T1DM had an HR of 1.73 (95% CI: 1.65–1.82) for depression, while females had an HR of 1.52 (95% CI: 1.44–1.60). Similarly, for sleep disorders, individuals with T1DM exhibited slightly higher risks compared to those with T2DM. In females, the risk was notably higher, with an HR of 1.05 (95% CI: 1.00–1.10) compared to T2DM.

**Table 3:** Subgroup analysis of sex-stratified risk of depression and sleep disorders among patients with type 1 diabetes mellitus, type 2 diabetes mellitus, and non-diabetic controls.

	T1DM	T2DM	Non-DM
*Depression*			
Male			
Incidence rate*	3.13 (2.99–3.28)	2.56 (2.47–2.65)	1.45 (1.40–1.49)
aHR	1.73 (1.65–1.82)	1.50 (1.44–1.57)	Reference
aHR	1.15 (1.10–1.21)	Reference	0.67 (0.64–0.70)
Female			
Incidence rate*	4.67 (4.48–4.87)	4.01 (3.89–4.13)	2.62 (2.56–2.69)
aHR	1.52 (1.44–1.60)	1.33 (1.28–1.39)	Reference
aHR	1.14 (1.08–1.20)	Reference	0.75 (0.72–0.78)
*Sleep disorder*			
Male			
Incidence rate*	5.83 (5.62–6.05)	5.39 (5.25–5.53)	3.00 (2.93–3.06)
aHR	1.58 (1.51–1.65)	1.52 (1.47–1.58)	Reference
aHR	1.03 (0.99–1.08)	Reference	0.66 (0.63–0.68)
Female			
Incidence rate*	8.15 (7.87–8.44)	7.45 (7.26–7.64)	4.73 (4.64–4.83)
aHR	1.38 (1.32–1.45)	1.32 (1.27–1.37)	Reference
aHR	1.05 (1.00–1.10)	Reference	0.76 (0.73–0.79)

aHR = adjusted hazard ration (adjusted by age, sex, and year of diagnosis), DM = diabetes mellitus, T1DM = type 1 diabetes mellitus, T2DM = type 2 diabetes mellitus.

* Incidence rate, per 1000 person-months.

Table 4 presents the age-stratified risk of depression and sleep disorders among patients with T1DM and T2DM, compared to non-diabetic controls. The data is divided into 4 subgroups: <20, 20–40, 40–65, and ≥65. For depression, individuals with diabetes consistently exhibited higher incidence rates across all age categories compared to non-diabetic controls. After adjusting for confounding factors, individuals with T1DM consistently displayed elevated risks of depression compared to those with T2DM and non-diabetic controls across all age groups. Notably, the risk was particularly pronounced in the older age groups, with individuals aged 65 and above showing the highest risk, with an HR of 1.64 (95% CI: 1.55–1.73). However, for sleep disorders, while individuals with diabetes exhibited elevated risks across all age categories, the difference in risk between T1DM and T2DM was not statistically significant.

**Table 4:** Subgroup analysis of age-stratified risk of depression and sleep disorders among patients with type 1 diabetes mellitus, type 2 diabetes mellitus, and non-diabetic controls.

	T1DM	T2DM	Non-DM
*Depression*			
Age < 20			
Incidence rate*	1.85 (1.58–2.18)	1.84 (1.63–2.08)	1.17 (1.06–1.29)
aHR	1.70 (1.35–2.14)	1.67 (1.41–1.97)	Reference
aHR	1.02 (0.81–1.29)	Reference	0.60 (0.51–0.71)
Age 20–40			
Incidence rate*	3.39 (3.11–3.70)	2.59 (2.42–2.77)	1.83 (1.73–1.93)
aHR	1.67 (1.48–1.88)	1.36 (1.24–1.50)	Reference
aHR	1.23 (1.09–1.38)	Reference	0.74 (0.67–0.81)
Age 40–65			
Incidence rate*	3.83 (3.67–4.00)	3.22 (3.12–3.32)	2.14 (2.08–2.19)
aHR	1.59 (1.51–1.67)	1.39 (1.33–1.44)	Reference
aHR	1.15 (1.09–1.21)	Reference	0.72 (0.69–0.75)
Age ≥ 65			
Incidence rate*	4.65 (4.40–4.91)	3.89 (3.73–4.05)	1.99 (1.92–2.07)
aHR	1.67 (1.58–1.77)	1.46 (1.39–1.53)	Reference
aHR	1.15 (1.09–1.22)	Reference	0.69 (0.65–0.72)
*Sleep disorder*			
Age < 20			
Incidence rate*	3.49 (3.08–3.94)	2.90 (2.63–3.20)	1.68 (1.55–1.83)
aHR	1.82 (1.50–2.20)	1.76 (1.53–2.03)	Reference
aHR	1.03 (0.86–1.25)	Reference	0.57 (0.49–0.65)
Age 20–40			
Incidence rate*	5.77 (5.37–6.20)	5.25 (4.99–5.53)	3.78 (3.64–3.94)
aHR	1.39 (1.26–1.53)	1.35 (1.26–1.45)	Reference
aHR	1.03 (0.93–1.13)	Reference	0.74 (0.69–0.80)
Age 40–65			
Incidence rate*	6.68 (6.45–6.92)	6.06 (5.91–6.22)	4.10 (4.02–4.18)
aHR	1.39 (1.33–1.46)	1.34 (1.29–1.39)	Reference
aHR	1.04 (0.99–1.09)	Reference	0.75 (0.72–0.78)
Age ≥ 65			
Incidence rate*	9.04 (8.65–9.46)	8.43 (8.16–8.70)	3.69 (3.59–3.80)
aHR	1.64 (1.55–1.73)	1.58 (1.51–1.65)	Reference
aHR	1.04 (0.98–1.10)	Reference	0.63 (0.61–0.66)

aHR = adjusted hazard ration (adjusted by age, sex, and year of diagnosis), DM = diabetes mellitus, T1DM = type 1 diabetes mellitus, T2DM = type 2 diabetes mellitus.

* Incidence rate, per 1000 person-months.

## 4. Discussion

This study demonstrated a significant association between diabetes mellitus and the risk of depression and sleep disorders in a large cohort of Taiwanese patients. Our findings underscore the substantial burden of mental health disorders among diabetic patients, revealing consistently elevated risks of depression and sleep disorders across different age and sex strata. Notably, individuals with T1DM exhibited a particularly heightened vulnerability to depression. Furthermore, while the risk of sleep disorders was elevated in both T1DM and T2DM groups compared to non-diabetic controls, differences between T1DM and T2DM were less pronounced.

While our study demonstrated the association between depression, sleep disorders, and diabetes, it is important to note baseline differences, particularly in the prevalence of hypertension, cardiovascular diseases, and the use of benzodiazepines. Despite our meticulous efforts to match cases and controls, these discrepancies persisted and may have influenced our observed outcomes. These variations in baseline characteristics could reflect underlying differences in health status, medication use, and comorbidity burden among the study cohorts, potentially confounding our results. However, it is noteworthy that our findings are in line with existing literature, which consistently reports a higher prevalence of depression among patients with both type 1 and type 2 diabetes.^[24]^

Moreover, this study showed a slightly elevated risk of depression in patients with T1DM compared to those with T2DM, with a HR of 1.11 (95% CI: 1.07–1.16). This finding is consistent with the distinct challenges faced by T1DM patients, including the immediate initiation of exogenous insulin therapy upon diagnosis, lifelong disease management, and heightened psychological distress, particularly among younger age groups.^[25]^ Conversely, T2DM patients, often initiating treatment with lifestyle modifications and oral antidiabetic medications, may experience a less immediate impact on mental well-being, given the older age at diagnosis and prevalence of comorbidities.^[26]^ Interestingly, our study did not find a significantly increased risk of sleep disorders in T1DM patients (HR: 1.01, 95% CI: 0.97–1.04).

The relationship between age and the risk of depression and sleep disorders differs between T1DM and T2DM (table 4). In T1DM, the risk of depression remains consistently elevated across all age groups, indicating a stable association regardless of age. However, in T2DM, there is a notable decreasing trend in the risk of depression with increasing age, suggesting that older individuals with T2DM may face relatively lower risks of depression compared to younger counterparts. Similarly, for sleep disorders, the pattern is distinct between T1DM and T2DM. In T1DM, younger age groups exhibit a higher risk of sleep disorders, with the risk decreasing as age advances. Conversely, in T2DM, there is also a decreasing trend in the risk of sleep disorders with increasing age, indicating a similar pattern to depression.

Previous studies have established a bidirectional relationship between diabetes mellitus and depression, with each condition exacerbating the other’s severity and prognosis.^[5]^ Our study contributes to this understanding by revealing a particularly heightened vulnerability to depression among individuals with T1DM. This finding highlights the need for targeted mental health screening and management strategies within diabetes care protocols, especially for patients with T1DM who may be at increased risk of developing depression.^[27,28]^

Additionally, this study indicates that the risk of sleep disorders is elevated in both T1DM and T2DM groups compared to non-diabetic controls. While this association has been documented previously,^[29,30]^ our study adds nuance by demonstrating that the differences in risk between T1DM and T2DM were less evident. This can be attributed by various physiological and psychological mechanisms. For sleep disorders, both T1DM and T2DM patients experience similar risks due to several shared factors. Blood glucose fluctuations in both types of diabetes can disrupt sleep, with nocturnal hypoglycemia or hyperglycemia affecting sleep quality. Diabetes-related complications such as neuropathy, which causes pain and discomfort, can also impair sleep in both groups. Furthermore, both T1DM and T2DM patients may experience frequent urination due to polyuria, a common symptom in diabetes that disrupts sleep. These shared disruptions can lead to a similar prevalence of sleep disorders among T1DM and T2DM patients, explaining the lack of significant difference in this aspect.^[30]^

The strengths of our study lie in its comprehensive methodology. Firstly, the utilization of a nationwide population-based dataset ensures a large and diverse sample, enhancing the generalizability of our findings to the broader Taiwanese population. Secondly, our study spanned an extended period, allowing for a longitudinal analysis of the relationship between diabetes mellitus and mental health outcomes, providing valuable insights into temporal trends and trajectories. Additionally, the meticulous matching of cases and controls, along with the adjustment for confounding variables, enhances the internal validity of our results. Finally, the inclusion of a wide range of potential confounders and comorbidities in our analyses ensures a thorough examination of the association between diabetes and mental health outcomes.

Despite its strengths, our study has several limitations that should be acknowledged. Firstly, as with any observational study, we cannot establish causality between diabetes mellitus and mental health outcomes due to the potential for unmeasured confounding factors. Secondly, like any large administrative database, the NHIRD has certain limitations that must be considered when interpreting our study results, including data accuracy. The NHIRD uses ICD-9-CM and ICD-10-CM coding systems for disease diagnoses, which carry a potential risk of coding errors or misclassification. To address this, we refer to data validation studies. For instance, the sensitivity and positive predictive value (PPV) for diabetes in the NHIRD have been reported as 90.9% and 92.0%, respectively.^[31]^ However, the validation of depression diagnoses in administrative data indicates a lower accuracy, with a sensitivity of 61.4% and a PPV of 69.7%.^[32]^ Therefore, the diagnosis of depression may have lower diagnostic accuracy compared to diabetes. Thirdly, although we adjusted for various confounders, residual confounding may still exist, which could influence the observed associations. Additionally, the lack of detailed clinical information, such as disease severity or treatment adherence, limits our ability to explore potential mechanisms underlying the observed associations. Lastly, our study was conducted in the Taiwanese population, and thus, the generalizability of our findings to other populations may be limited. However, the physiological mechanisms linking diabetes and depression are likely universal. Cultural differences in the perception and management of diabetes and mental health may influence depression and sleep disorder prevalence. Studies in the United States and Europe, as well as multicenter cohort studies like the Diabetes Attitudes, Wishes and Needs (DAWN) study, have reported similar associations, suggesting our findings may be applicable to other contexts.^[33]^

While our study provides valuable insights into the association between diabetes mellitus and mental health outcomes, there are several prioritized avenues for future research. High priority should be given to prospective cohort studies to examine the temporal relationship between diabetes diagnosis and mental health outcomes over time. Randomized controlled trials should also be prioritized to evaluate the effectiveness of integrated care models that combine diabetes management with mental health support. Additionally, medium priority should be assigned to cross-sectional and longitudinal studies investigating underlying mechanisms, such as glucose control, socioeconomic status, and frequency of insulin injections. Finally, randomized controlled trials and observational studies assessing the effectiveness of psychoeducation programs and pharmacological interventions in managing depression, sleep disorders, and other mental health concerns among individuals with diabetes are also important. These research designs will help to address the remaining gaps and advance our understanding of the complex interplay between diabetes and mental well-being.

In conclusion, our study highlights the significant association between diabetes mellitus and mental health outcomes, particularly depression and sleep disorders, in a large cohort of Taiwanese patients. These findings underscore the critical importance of integrating mental health screening and management into routine diabetes care protocols. The heightened vulnerability observed, especially among patients with T1DM, necessitates proactive measures by healthcare providers to address mental health concerns effectively. Moreover, our results emphasize the need for continued research and clinical efforts to better understand and mitigate the impact of mental health issues in diabetic populations, thereby enhancing overall diabetes care and public health outcomes.

## Author contributions

**Conceptualization:** Shih-Chang Lo, Edy Kornelius.

**Data curation:** Jing-Yang Huang.

**Formal analysis:** Yi-Sun Yang, Jing-Yang Huang, Chien-Ning Huang.

**Investigation:** Jing-Yang Huang.

**Methodology:** Yi-Sun Yang, Shih-Chang Lo, Edy Kornelius.

**Resources:** Chien-Ning Huang.

**Software:** Jing-Yang Huang.

**Supervision:** Chien-Ning Huang.

**Visualization:** Jing-Yang Huang.

**Writing – original draft:** Meng-Han Ni, Edy Kornelius.

**Writing – review & editing:** Edy Kornelius.

## Supplementary Material




