# Supplementary material for: The association of depression and sleep disorders in patients with type 1 diabetes in Taiwan
Source: Medicine (Baltimore). 2024 Jul 19;103(29):e38969. doi: 10.1097/MD.0000000000038969 (PMC11398819; doi:10.1097/MD.0000000000038969)
Supplement: Supplementary file 1 [file medi-103-e38969-s001.docx]

**Supplemental table 1**

Type 1 diabetes mellitus (T1DM)

- ICD-9-CM: 250.x1, 250.x3
- ICD-10-CM: E10

Type 2 diabetes mellitus (T2DM)

- ICD-9-CM: 250.xx, excluding 250.x1, 250.x3
- ICD-10-CM: E11-E13

**Co-morbidities and co-medications**

| Co-morbidities | ICD-9 codes | ICD-10 codes |
| --- | --- | --- |
| Asthma | 493 | J45 |
| Allergic rhinitis | 477 | J30 |
| Atopic dermatitis | 691, 692 | L23-L25 |
| Hypertension | 401-405 | I10, I11, I13, I15, I16, I87.3, I97.3, O10, O11, O13, O16 |
| Cardiovascular disease | 410-414 | I20-I25 |
| Heart failure | 398.91, 402.x1, 404.x1, 404.x3, 428 | I09.81, I11.0, I13.0, I50 |
| Rheumatic disease | 446.5, 710, 714, 725 | M05, M31.6, M32, M33, M34, M35.3 |
| Malignancy | 140-208 | C00-C96 |
| Seizure disorder | 345, 649.4 | G40 |
| End-stage renal disease | 585 | N18.4, N18.5, N18.6 |
| Ischemic stroke | 433-435 | I63, I65, I66, G45, G46 |
| Hemorrhage stroke | 430-432 | I60-I62 |
| ADHD | 314 | F90 |
| Autism | 299.0 | F84.0 |

**Study outcomes**

Depression

- ICD-9: 296, 300,309,311
- ICD-10: F30-F34, F40-F45

Sleep disorders

- ICD-9: 327, 347, 307.4, 770.8, 780.5, V69.4
- ICD-10: F51, G47, Z72.8
